# Voxel-based evaluation of hemorrhage risk in brain biopsies

**DOI:** 10.1007/s10143-025-04132-6

**Published:** 2026-01-30

**Authors:** Mykola Gorbachuk, Aldo Spolaore, Eliane Weinbrenner, Sophie Wang, Kathrin Machetanz, Marcos Tatagiba, Georgios Naros

**Affiliations:** 1 Department of Neurosurgery and Neurotechnology, Eberhard Karls University, Tuebingen, Germany; 2 Department of Neurosurgery and Neurotechnology, Eberhard Karls University Hospital, Hoppe-Seyler-Str. 3, 72076 Tuebingen, Germany

**Keywords:** Stereotactic brain biopsy, Hemorrhage, Robot-assisted surgery, Frame-less stereotactic surgery, Stereotaxy

## Abstract

While technological progress increases precision and reduces invasiveness of stereotactic brain biopsies (BB), biopsy related hemorrhage (BBH) is still a key risk. This study identifies risk factors and uses voxel-based lesion symptom mapping (VLSM) to analyse the spatial distribution of BBH. We analyzed 450 frame-based and robotic-assisted BB. Patients’ preoperative MR and postoperative CT imaging were registered and normalized to the standard MNI space enabling volumetry and inter-subject comparison of BBH location. Binary logistic regression analysis was performed to determine significant BBH predictors. Additionally, we performed VLSM to evaluate the exact spatial profile of BBH in relation to the functional outcome. BBH was noted radiographically in 80 cases (18%) with a mean volume of 1.9 ± 19.0 ml. 19/450 (4%) of all BB presented symptomatic BBH characterized mainly by sensorimotor deficits (13/450,3%) and/or reduced vigilance (5/450,1%). 7/450 (2%) cases required surgical evacuation of BBH and 10/450% (2%) patients suffered from persistent neurological deficits. High-grade glioma, patient age and target location were main BBH predictors. VLSM determined frontal trajectories targeting deep-seated lesions in the basal ganglia to be significantly associated with a higher BBH risk. BBH within the posterior aspect of the basal ganglia, insula and capsula interna emerged as significant predictors for neurological deterioration after surgery. While asymptomatic hemorrhages are quite common after brain biopsies, neurological deterioration is rare. BBH risk is influenced by both spatial factors and non-spatial factors. BB targeting basal ganglia were linked to a higher risk of hemorrhage, particularly symptomatic BBH with somatosensory deficit.

## Introduction

Despite recent advances in neuroimaging, an accurate non-invasive diagnosis of intracranial expanding lesions has not yet been achieved. The incorporation of molecular and morphological features in WHO classification of brain tumors (2021) has boosted the need for molecular processing of tissue samples in brain lesions, increasing the incidence of brain biopsies (BB) [28, 38]. At the same time, technological evolution (e.g. the introduction of robotics) has simplified the process of sampling tumor tissue by decreasing invasiveness and increasing time efficiency [19, 20, 26]. Nevertheless, hemorrhage is still a significant complication of BB. The risk of asymptomatic hemorrhage varies between 8% and 60% [12, 15, 24], while symptomatic hemorrhages occur in 1–10% of cases [2, 14, 23, 32]. Several attempts have been made to determine clinical, surgical and imaging predictors of BB-related hemorrhages (BBH) resulting in mainly inconclusive results. Several surgeon-related [16, 43, 45], patient-related [10, 22–24] or tumor-related [15, 22, 24, 32] risk factors have been described including surgeon’s experience [16], trajectory planning [43, 45], higher patient age [22] and comorbidities (e.g. diabetes mellitus, reduced platelet count, prolonged prothrombin time) [10, 23, 24] and intraoperative hypertension [17].

In summary, vascularity along the stereotactic corridor and within the lesion could be considered the main predictor for BBH. Vascularity depends on the histological characteristics of the lesion and the lesion location. High-grade glioma, known for their high vascularity [27, 31], have been shown to have a higher BBH risk [14, 17, 23, 24]. BB within the basal ganglia, a highly vascularized brain region, are also associated with a greater BBH hazard [12, 17, 23]. In line, intratumoral susceptibility (as derived from susceptibility-weighted imaging, SWI) indicates higher BBH risk [9, 35]. In addition, trajectories intersecting structures at risk (such as vessel or sulcus) are known to be associated with BBH [44]. Independently, the BBH location predicts its clinical manifestation: BBH in eloquent areas have a higher rate of neurological deficits [21, 23, 30, 39].

In our opinion, the role of biopsy location is underestimated in literature. Voxel-based lesion symptom mapping (VSLM) offers a methodological framework to study the spatial distribution of BBH. VSLM enables rater-independent, inter-subject statistical analysis of spatial relationships between lesions and outcome predictors. VSLM has been widely applied focusing on stroke and other brain tumors [7, 8, 34, 42]. Researchers have used VLSM to pinpoint loci where damage correlates with cognitive or motor impairments, achieving a regional specificity unattainable with older methods [40, 47]. However, there are no studies on VSLM evaluating the relationship between BB targets, the occurrence of BBH and the resulting clinical outcome. The present study aims to evaluate the location of BBH applying unbiased VSLM.

## Methods

### Patients

This retrospective study enrolled 450 consecutive patients undergoing frame-based or robot-assisted brain biopsy between 03/2009 and 02/2021 in the Department of Neurosurgery and Neurotechnology of the University Hospital of Tuebingen, Germany. The study was approved by the local ethics committee (No. 015/2020BO2) and performed in accordance to the Declaration of Helsinki. Due to its retrospective character, there was no clinical trial registration (Clinical trial number: not applicable). The inclusion criteria were (i) a lesion affecting the brain, cerebellum or brain stem, (ii) the presence of a preoperative high resolution MR imaging and (iii) the presence of a postoperative CT. Individual decision for biopsy was made during institutional multidisciplinary patient management conferences.

### Standard operating procedure (SOP) for intracranial biopsies

Patients underwent preoperative high resolution T1-weighted (double) contrast-enhanced MPRAGE, as well as FLAIR and T2 MRI. The entry (EP) and target point (TP) of the stereotactic trajectory were planned using BrainLab (iPlanet 3.1, Brainlab, Munich, Germany) or the ROSA^®^ (ROSA One^®^, Zimmer Biomet, Warsaw, USA) software following general stereotactic principles: (i) avoiding sulci or transventricular trajectories and the unnecessary violation of eloquent areas, (ii) avoiding conflicts with the stereotactic frame, (iii) shortening the trajectory lengths [6, 18, 29, 48].

In frame-based stereotaxy, the stereotactic frame (CRW, Radionics Inc., Burlington, Massachusetts, USA) was usually fixed at patient’s skull in local anesthesia at bedside. Subsequently, a CT was acquired with a localizer fixed to the stereotactic frame. The planning software (iPlanet 3.1, Brainlab, Munich, Germany) provided the coordinates which were manually applied to the stereotactic arc during surgery.

All robot-assisted biopsies were performed with the assistance ROSA one^®^ (Zimmer-Biomet, Warsaw, USA) [26]. Patient-to-robot registration is performed either by (i) pair-point registration (e.g., bone fiducial registration, *BFR*) or (ii) laser surface registration (*LSR*) [20]. For *BFR*, five bone fiducials (WayPoint™, FHC, Bowdoin, USA) were placed on the day of surgery at bedside under local anesthesia just before CT scanning.

After positioning, the patient’s head was fixed by the stereotactic frame or by a Mayfield skull clamp. Depending on the trajectory characteristics, surgeons decided on the surgical approach: i.e., a 1.4 cm burr hole trepanation (Elan 4, B.Braun, Freiburg, Germany) or a 2.1 mm twist drill trepanation (Acculan 4, B.Braun, Freiburg, Germany). Biopsies were taken in several quadrants along the trajectory with a biopsy cannula (1.8 × 250 mm, BrainPro, Pajunk, Geisingen, Germany). A frozen section examination was performed. A postoperative CT scan was performed within 24 h after the surgery.

### Clinical data

Demographics, the occurrence of symptomatic hemorrhages and permanent morbidity were systematically analyzed. Neurological deficits that endured over one year were defined as persistent. Histological results were collected to evaluate the diagnostic yield (*DY*) [19].

### Imaging data processing

**Fig. 1:**
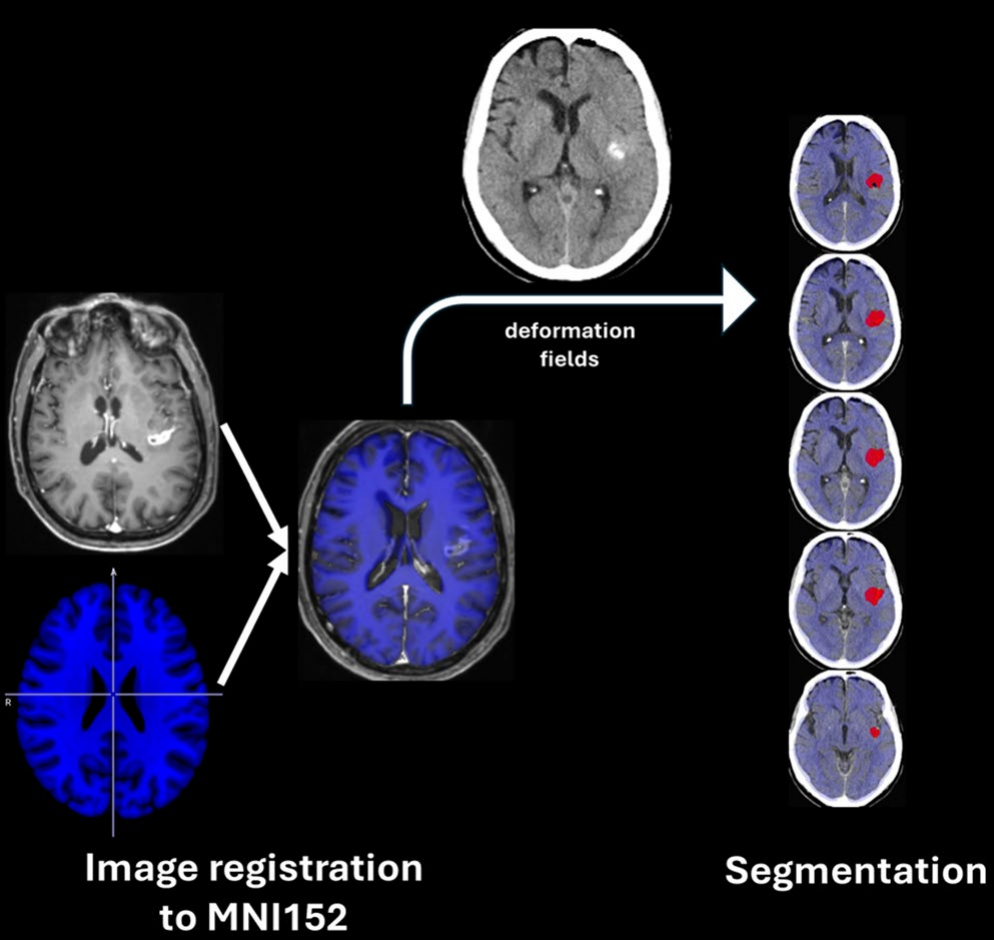
Workflow for voxel-based lesion mapping. Patients’ individual high-resolution preop-MRI was registered to a 1-mm isotropic, high-resolution, T1-weighted MRI template provided by the MNI (the MNI152). The resulting deformation fields were passed to the individual low-resolution postop-CT. Brain biopsy related hemorrhage (BBH) was semi-automatically segmented and flipped to project to the right hemisphere. This information was used for the voxel-wise lesion symptom mapping (VLSM) analysis

Statistical Parametric Mapping Software version 12 (SPM12, Institute of Neurology, University College London, London, UK; https://www.fil.ion.ucl.ac.uk/spm) [11] and MATLAB (R2024a, MathWorks, Natick, MA, USA) were used to register and normalize patient’s preoperative MR images to a standard brain template (MNI152; Montreal Neurological Institute, McGill University, Montreal, Quebec, Canada). The resulting deformation fields were saved for further analysis. Entry point *(EP)* and target point (*TP)* coordinates -provided by the planning software were transferred to the MNI152 space with the individual deformation fields. Normalized (*X*,* Y*,*Z*) coordinates of the *EP* and *TP* were used to calculated the length of the trajectory and to reconstruct a trajectory mask (diameter of 10 voxel). The trajectory masks were flipped to project to the right hemisphere and saved for further analysis. In addition, we compared the normalized *TP* coordinates with the *AAL* atlas [36] to classify the anatomical *TP* location. This classification was visually reviewed and corrected for regions unrecognized by the *AAL* atlas (e.g., white matter tracts, brainstem, cerebellum). Postoperative CT scans were fused to the preoperative MRI and the previous calculated deformation fields were applied to the CT scan transferring it to the MNI152 space (Fig. 1). The BBH was semi-automatically segmented using a fast marching method implemented in MATLAB (*imsegfmm.m*). The segmentation was performed and reviewed by two neurosurgeons. Manual corrections of the tumor mask were made in MRIcron (https://www.nitrc.org/projects/mricron/). The individual BBH volume was noted. BBH volumes > 0.5 ml were considered significant [44, 45]. The BBH masks were flipped to project to the right hemisphere and saved for further analysis.

### Voxel-based symptom lesion mapping (VSLM)

VSLM was based on an univariate linear regression model and one-tail t-statistics as implemented in SPM12 (https://www.fil.ion.ucl.ac.uk/spm) and NiiStat (https://www.nitrc.org/projects/niistat). VLSM enables evaluation of the relationship of a predictor (e.g., BBH occurrence) to a specific outcome (e.g., postoperative paresis) at individual voxels (i.e., voxel-vice) [3]. Categorial outcome variables (e.g., histology) were incorporated after dummy coding (0: reference group; 1: observational group). Only voxels with signal in at least 5 participants were included in the analysis. For trajectory masks, this approach resulted in 457,114 analyzed voxel; for BBH masks, 16,142 voxel were analyzed. Multiple comparison correction was based on the Bonferroni method.

### Statistical analysis

All statistical tests were performed using Matlab (R2019a, Mathworks Inc., Natick, MA, USA) and SPSS (IBM SPSS Statistics for Windows, version 22.0. Armonk, NY: IBM Corp.). For comparison of categorical data, we used a chi-squared test (X² test). Group comparisons of metric variables were based on non-parametric Kruskal-Wallis Tests. For correlation analysis of two metric variables, we applied a Person’s correlation. For multivariate analysis of multiple predictors on the occurrence of BBH, we applied a binary logistic regression. p-values < 0.05 were considered significant. Unless otherwise specified, data is presented as mean ± standard deviation (SD).

**Table 1:** Patients’ characteristics. Patients were grouped based on the presence (***BBH***) or absence (***no BBH***) of postoperative brain biopsy-related hemorrhages (*BBH*). These groups were statistically compared, *p* < 0.05 is considered significant and highlighted. Misc: miscellaneous. *incl. thalamus, ** inflammatory lesions with or without concomitant demyelination

	Total	BBH	no BBH	
No. of patients *robot-assisted* *frame-based*	450 230 (51%) 220 (49%)	80 40 40	370 190 180	X²=0.07; *p* = 0.792
Age (mean ± SD) Gender (f: m)	58.3±19.2 210:240	64,5±16.6 41:39	56,9±19.4 199:171	H = 12.22; *p* < 0.001 X²= 0.17; *p* = 0.680
Trajectories * Number* * length (mm)* *Target locations* * frontal* * parietal* * temporal* * occipital* * insular* * cerebellum* * brainstem* basal ganglia* * misc.*	472 49.7±19.3 127 (28%) 62 (14%) 61 (14%) 9 ( 2%) 22 ( 5%) 40 ( 9%) 12 ( 3%) 114 (25%) 3 ( 1%)	84 51,4±17,3 17 (3,8%) 9 (2%) 11 (2,4%) 2 (0,4%) 2 (0,4%) 6 (1,3%) 2 (0,4%) **30 (6**,**7%)** 1 (0,2%)	388 49,2±19.7 110 (24,2%) 53 (12%) 50 (11,6%) 7 (1,6%) 20 (4,6%) 34 (8,7%) 10 (2,6%) **84 (18**,**3%)** 2 (0,4%)	X²= 0.68; *p* = 0.712 H= 1.47; *p* = 0.220 X²= 2.33; *p* = 0.127 X²= 0.52; *p* = 0.469 X²= 0.00; *p* = 0.955 X²= 0.12; *p* = 0.725 X²= 1.19; *p* = 0.274 X²= 0.23; *p* = 0.630 X²= 0.01; *p* = 0.919 ***X²= 7.61; p = 0.006*** X²= 0.50; *p* = 0.482
Histology * HGG* * LGG* * metastasis* * lymphoma* inflammation** * abscess* * others* * inconclusive*	214 (47%) 87 (20%) 18 ( 4%) 62 (14%) 45 (10%) 8 ( 2%) 11 ( 2%) 4 ( 1%)	47 (10,5%) 7 (1,5%) 6 (1,3%) 9 (2%) 10 (2,2%) 1 (0,2%) 11 (2%) 4 (1%)	167 (37,1%) 80 (18,5%) 12 (2,7%) 53 (12%) 35 (7,8%) 7 (1,8%) 0 0	***X²= 4.89; p = 0.027*** **X²= 6.98; p = 0.008** X²= 3.10; *p* = 0.078 X²= 0.52; *p* = 0.469 X²= 0.67; *p* = 0.411 X²= 0.16; *p* = 0.694 X²= 0.87; *p* = 0.350 X²= 2.43; *p* = 0.118

## Results

### Clinical characteristics

This retrospective study evaluated 450 patients undergoing conventional frame-based (*n* = 220) or robot-assisted (*n* = 230) BB. Most biopsies aimed for targets within the frontal lobe and the basal ganglia with a mean trajectory length of 50 ± 19 [15–114] mm. Overall, the diagnostic yield was 99% with high-grade and low-grade glioma as well as intracerebral lymphoma representing the most frequent histologic entities. Clinical data is summarized in Table 1.

Significant BBH was noted radiographically in 80/450 (18%) cases with a median BBH volume of 1.9 ± 19.0 [0.5-111.9] ml. In 61/80 (76%) cases BBH were asymptomatic. In 51/80 (64%) cases BBH was located within the lesion, in 10/80 (13%) cases BBH was detected extralesionally along the trajectory and in 19/80 (24%) cases BBH occurred in both the lesional and extralesional part of the trajectory. An overview of BBH characteristics is given in Table 2.

Only 19/450 (4%) biopsies resulted in a symptomatic BBH characterized by sensorimotor deficits 13/450 (3%) and/or reduced vigilance 5/450 (1%). 7/450 (2%) cases necessitated revision surgery (i.e., BBH evacuation) and 10/450 (2%) patients suffered from persistent neurological deficits. In one case of an elderly multimorbid patient with a significant space occupying BBH and rapid clinical deterioration, palliative care was initiated resulting in an overall mortality of 1/450 (0.2%).

**Table 2:** Characteristics of brain biopsy-related hemorrhage

Hemorrhage Features *volume of hemorrhage (ml)* *asymptomatic hemorrhage* *symptomatic hemorrhage* * sensorimotor deficits* * aphasia* * reduced vigilance* *hemorrhages in relation to trajectory* * intralesional* * extralesional* * intra- and extralesion* *revision surgery* *permanent deficits* *mortality*	1.9 ± 18.9 [0.5-111.8] 61 (14%) 19 (4%) 13 (3%) 1 (0.2%) 5 (1%) 51 (11%) 10 (2%) 19 (4%) 7 (2%) 10 (2%) 1 (0.2%)

### Predictors of BBH occurrence and BBH volumes

On an univariate level, the occurrence of BBH was associated with high-grade glioma (HGG) (X²=4.89, *p* = 0.027), target location within the basal ganglia (BG) (X²=7.61, *p* = 0.006), and older patient AGE (H = 12.22, *p* < 0.001). Notably, low-grade glioma (LGG) had a significant lower risk of BBH (X²=7.00, *p* = 0.008). There was no association of BBH and patients’ sex (X²=0.17, *p* = 0.680), the length of the trajectory (H = 1.47, *p* = 0.226), the used stereotactic system (X²=0.07, *p* = 0.792) and other target locations. For multivariate analysis, we applied a binary logistic regression to the covariates (i.e., BG, AGE, HGG, LGG). A significant model (X²=24.49, *p* < 0.001, Nagelkerke’s R²=0.087) was detected with AGE (β = 0.022; *p* = 0.007) and BG location (β = 0.749; *p* = 0.005) as significant BBH predictors.

In contrast, BBH volume was not associated to patients’ age (*r* = 0.022, *p* = 0.845) or sex (H = 3.33, *p* = 0.680), the used stereotactic system (H = 0.02, *p* = 0.878), lesion histology (H = 8.56, *p* = 0.128), trajectory length (*r*=-0.082, *p* = 0.470) or target location (H = 14.83, *p* = 0.062). However, BBH volume determined the clinical manifestation. Symptomatic BBH had significantly larger median volumes than asymptomatic BBH (7.4 ± 34.4 ml and 1.4 ± 2.6 ml; H = 20.20, *p* < 0.001). BBH necessitating revision surgery had the largest volumes (21.5 ± 36.6 ml).

**Fig. 2:**
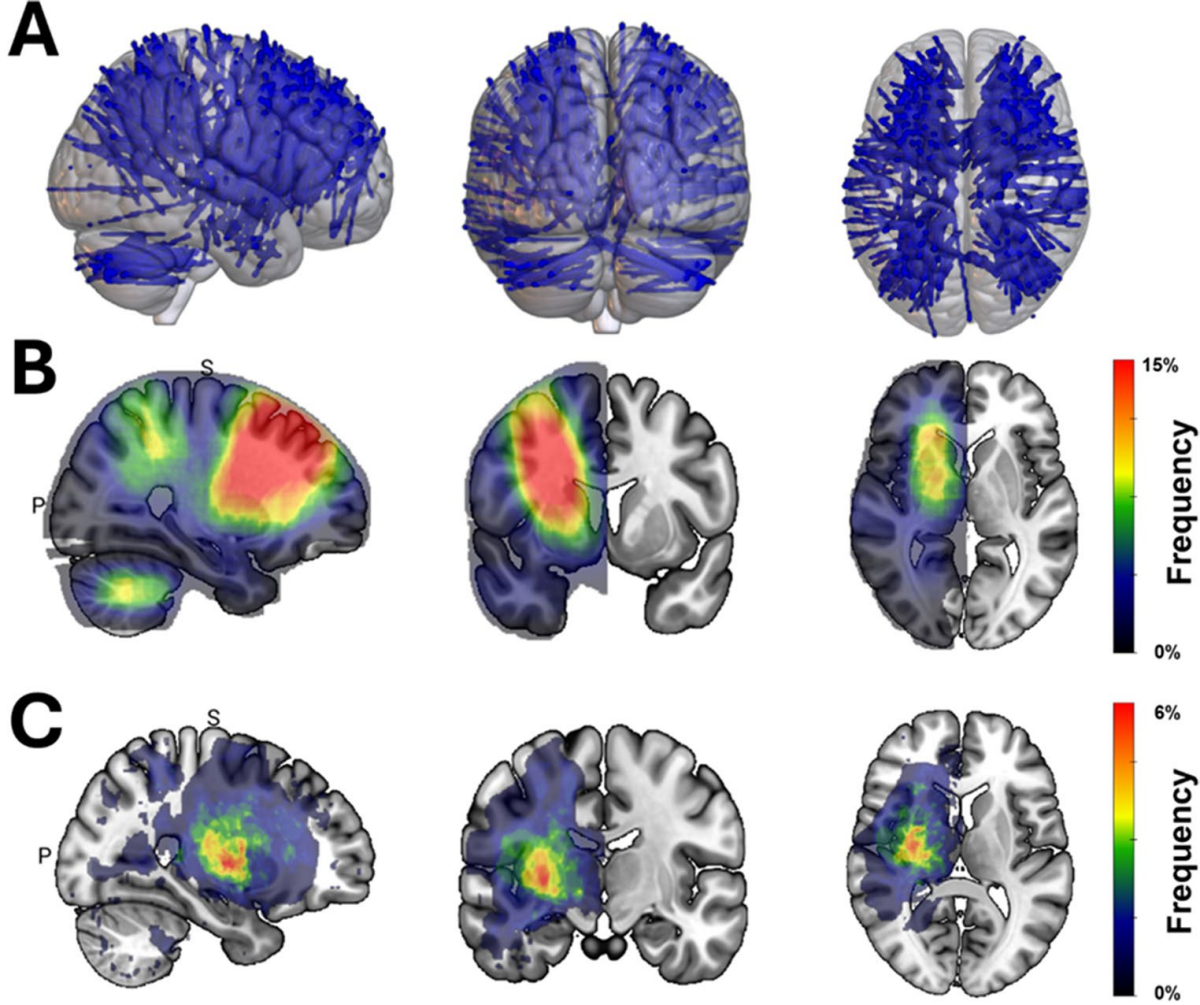
Spatial distribution of stereotactic brain biopsies. (**A**) Location of the evaluated biopsy trajectories depicted on right, posterior and superior view. (**B**) For statistical analysis, trajectories were mirrored to the right hemisphere. Color frequency maps of the biopsy trajectories (on a sagittal, coronal and axial view) indicated three main trajectory corridors (i.e., frontal, parietal and cerebellar). Notably, targets within the basal ganglia and insula were usually targeted via frontal trajectories. (**C**) Frequency maps of BBH (in a right, coronal and axial view) demonstrate a higher risk of BBH for biopsies within the basal ganglia

### Spatial distribution of BBH

The spatial normalization of the individual preoperative MR images enables the visualization and analysis of all trajectories in the common *MNI152* space (Fig. 2A). Entry and target point information was used to reconstruct a 3D trajectory mask which was mirrored to the right hemisphere. Overlapping trajectory masks uncovered three main corridors applied in the present BB cohort, i.e. frontal, parietal and transcerebellar. Most BB used a frontal approach, in particular for targets within the basal ganglia and insula (Fig. 2B). Notably, transcerebellar trajectories were only applicable in robot-assisted BB. The deformation field acquired from spatial normalization of the preoperative MRI was transferred to the postoperative CT scan. BBH were segmented for each patient and overlapped. This analysis demonstrated a predominance of BBH in the regions of the basal ganglia and the insula (Fig. 2C).

**Fig. 3:**
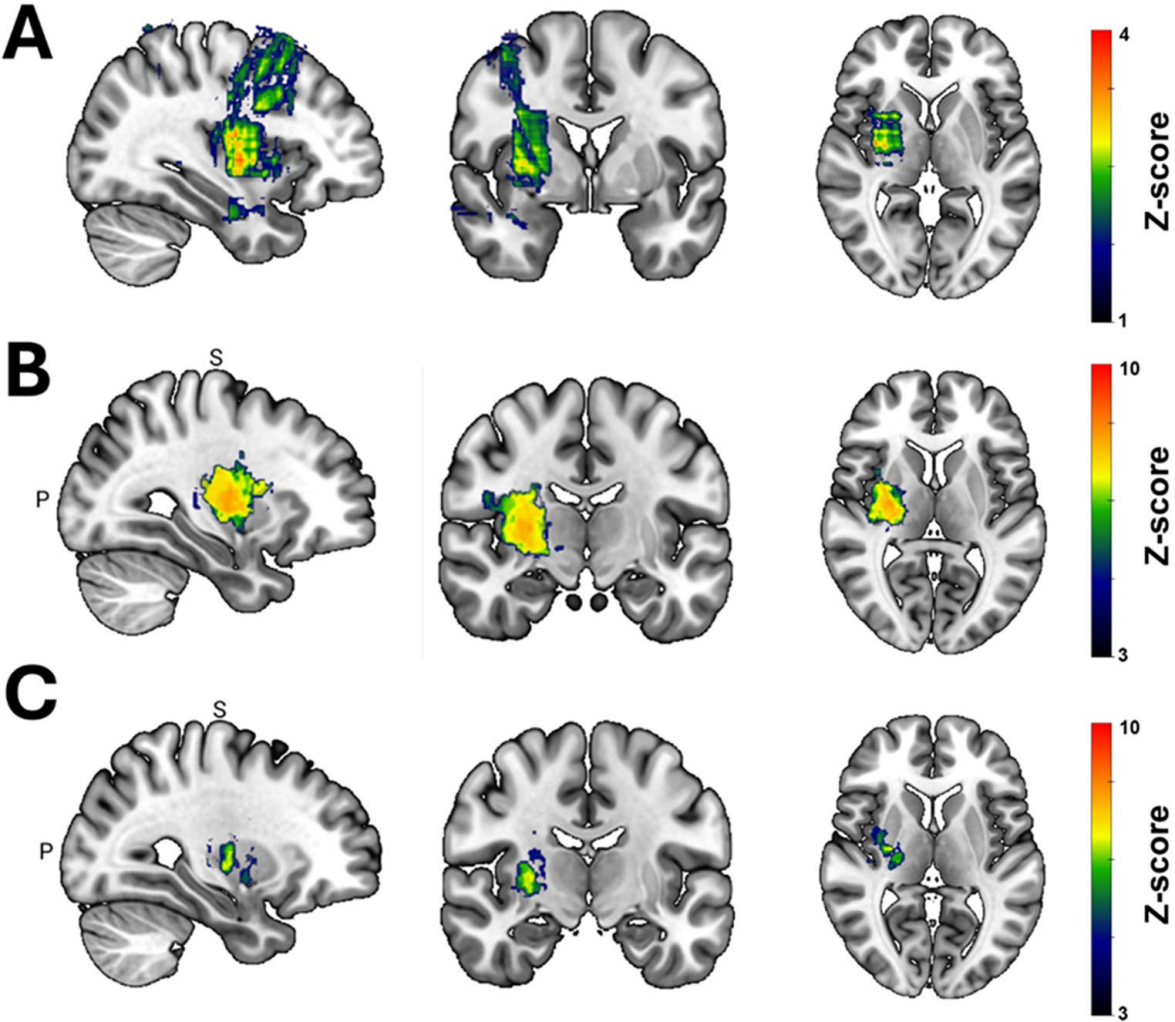
Spatial distribution of brain biopsy related hemorrhages (BBH). (**A**) Voxel-based symptom lesion mapping (VSLM) determined frontal trajectories targeting deep-seated lesions to be significantly associated with a higher risk for BBH. Z-scores are thresholded for *p* < 0.05 (*uncorrected*). (**B**) VLSM analyzing the relationship between BBH location and function outcome confirmed that BBH within the posterior aspect of the basal ganglia, insula and capsula interna are more likely to cause neurological deterioration (e.g. sensorimotor deficits, reduced vigilance and aphasia). Significant Z-scores are thresholded and corrected for multiple comparisons (*p* < 0.05; Bonferroni). (**C**) In particular, sensorimotor deficits are typical symptoms of BBH within the putamen, globus palidum and the posterior limb of the internal capsule. Significant Z-scores are thresholded and corrected for multiple comparisons (*p* < 0.05; Bonferroni)

VLSM was applied to evaluate the relationship between trajectory location (*predictor*) and the occurrence of BBH (*outcome*). This analysis indicated that frontal trajectories targeting deep-seated lesions were most likely to cause BBH (Fig. 3A). Moreover, we used VLSM to analyze the spatial relationship between BBH (predictor) and the occurrence of symptoms (*outcome*). Notably, BBH within the posterior aspect of the basal ganglia, insula and capsula interna emerged as significant predictors for neurological deterioration after surgery (Fig. 3B). This association was in particular evident when analysis was repeated for the occurrence of postoperative sensorimotor deficits. These deficits showed a strong correlation with BBH specifically within the posterior portions of the putamen, globus pallidus, and the posterior limb of the internal capsule (Fig. 3C).

**Fig. 4:**
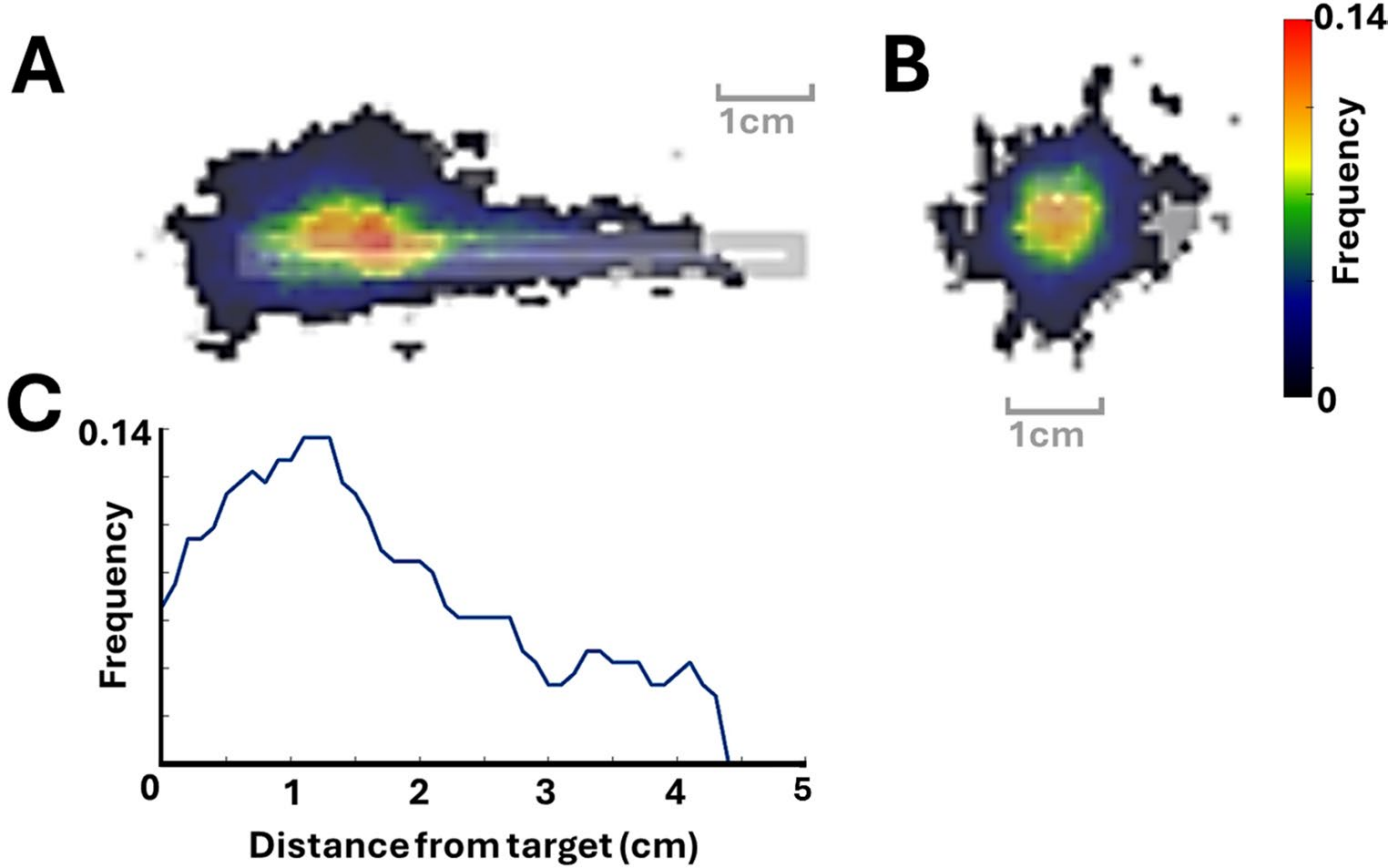
Spatial distribution of biopsy-related brain hemorrhages (BBH) along a standardized biopsy trajectory. To assess the spatial distribution of BBH events in regard to trajectory level, individual trajectory masks and hemorrhage segmentations were spatially normalized to a standardized biopsy trajectory of fixed length. (**A**) Heatmap showing the frequency of BBH along the length of the standardized trajectory, with the biopsy needle assumed to enter from the left and terminate at the target point on the right. (**B**) Cross-sectional heatmap perpendicular to the trajectory axis, centered at the target point, illustrating the lateral distribution of BBH around the target site. (**C**) Line plot showing the frequency of BBH as a function of distance from the target point (0 cm). The analysis demonstrates a higher hemorrhage risk at the distal end of the biopsy trajectory

To visualize BBH distribution along the biopsy trajectory, trajectory masks and bleeding segmentations were spatially normalized to a standardized trajectory of defined length. Frequency analysis revealed a predominance of biopsy-related hemorrhages at the target site of the trajectory (Fig. 4A-C).

## Discussion

The present study shows that asymptomatic BBH are relatively common in postoperative CT imaging. However, they rarely become symptomatic or necessitate intervention. We demonstrate that the BBH risk is affected by both spatial (i.e. target location) and non-spatial (i.e. patient and tumor characteristics) factors. In detail, the presence of HGG as well as the patient age are increasing the risk for BBH. In turn, target location at the level of the basal ganglia, insula and the posterior limb of the internal capsule is associated with a higher hemorrhage risk, in particular for symptomatic BBH with somatosensory deficits.

Our study identified an increase of asymptomatic BBH of approx. 14%, while symptomatic BBH occurred in approx. 4% of the cases. These numbers are in good accordance with literature which describes asymptomatic hemorrhage in 8–60% [12, 15, 24] and symptomatic hemorrhages in 1–10% of cases [14, 23, 32]. A striking variation of the occurrence of asymptomatic BBH in the literature could be explained by variable definition of significant BBH. In line with recent BBH studies [44–46], we have defined significant BBH by a volume of ≥ 0.5 ml. BBH of greater volume were associated with persistent neurological deficits and the necessity of surgical revision. Previous attempts to identify BBH predictors have highlighted surgeon- and patient-related risk factors. However, as most postoperative bleedings occur at the lesion sampling site [15, 24, 32], lesion entity and location of the target appear to be further important factors in understanding the occurrence of BBH.

In our survey HGG was a significant BBH predictor. This effect was independent of the spatial profile of these lesions. Malignant tumors [15, 17, 24, 32] have been shown previously to affect BBH hazard, as pathological angiogenesis within the tumor might contribute to BBH [5, 13, 35]. In contrast, according to our analysis LGG were associated with a lower BH risk. We hypothesize that the disruption of the blood–brain barrier and increased vascularity characteristic of HGG contribute to this higher risk. However, there is no evidence so far, that molecular glioma markers such as IDH mutation, 1p/19q codeletion, and MGMT promoter methylation affect BBH risk. Future studies exploring this association may provide valuable insights into the pathophysiology of BBH. In contrast to our findings, previous series have suggested a non-negligible rate of symptomatic biopsy-related hemorrhage in primary central nervous system lymphoma compared with other histotypes [4, 25, 41]. The limited number of CNS lymphoma cases in our cohort may partially explain this discrepancy. Future studies with larger cohorts will be necessary to clarify histotype-specific hemorrhagic risk and its interaction with anatomical risk factors.

In our opinion, the relationship between BB location and BBH occurrence remains underexplored in the literature. BB site could affect BBH risk due to several factors. First, the location might also affect the vascularity of the lesion. Tumors within the basal ganglia, a highly vascularized brain area, are well known to have a higher risk for BBH [12, 17, 23] as well as the thalamus [23] and the pineal region [10]. Second, stereotactic inaccuracy could contribute to the higher BBH risk in deep-seated tumors. In the present study, lesion location within the basal ganglia and the insula was an independent risk factor for BBH. As there was no association with the length of the trajectory or the stereotactic system (frame vs. robot), we do not expect stereotactic inaccuracy to be responsible for this finding. Moreover, BBH site is affecting the functional outcome. In the present study, BBH within the posterior basal ganglia were significant triggers of symptomatic hemorrhages with a higher risk for persistent deficits. The most common symptomatic manifestations were sensorimotor deficits (3%). These deficits showed a strong correlation with BBH specifically within the posterior portions of the putamen, globus pallidus, and the posterior limb of the internal capsule.

### Clinical implications

Despite its retrospective design, the present study provides clinically relevant insights that extend beyond descriptive risk reporting. By linking biopsy-related hemorrhage to neurological outcome at a voxel-wise level, VLSM enables anatomically informed trajectory planning. Rather than relying on broad anatomical labels, this approach provides spatially resolved risk information that may assist surgeons in comparing alternative trajectories and avoiding high-risk regions. Such outcome-linked spatial refinement has largely been absent from previous studies, which predominantly relied on region-based or categorical analyses. Beyond surgical planning, voxel-based risk maps may enhance preoperative patient counseling by enabling hemorrhagic risk to be communicated in a more individualized and anatomically grounded manner. Given that biopsy-related hemorrhage remains a major determinant of postoperative morbidity and patient anxiety [24], transparent, anatomy-informed counseling represents a meaningful clinical advance. Integration of voxel-wise spatial risk maps with established clinical variables (e.g., age, lesion depth) and histological features may enable multivariable risk stratification tools capable of estimating individualized hemorrhagic risk preoperatively. In this context, our results should be regarded as hypothesis-generating, representing an intermediate step toward prospective validation and individualized surgical planning rather than a definitive clinical decision algorithm. Our findings should also be interpreted in light of emerging non-invasive diagnostic alternatives. Advances in radiomics, artificial intelligence, and advanced MRI increasingly allow non-invasive predictions of glioma subtype and molecular status [1, 33]. While histological confirmation remains the clinical gold standard, such approaches may represent valuable adjuncts or alternatives in patients at highest risk of symptomatic biopsy-related hemorrhage, particularly for deep-seated basal ganglia lesions. Finally, the predominance of asymptomatic, radiographically confined hemorrhages raises questions regarding postoperative management. In patients with non-risky biopsy targets and an uneventful neurological examination, routine prolonged observation or mandatory postoperative imaging may be reconsidered reducing unnecessary radiation and costs [37].

The limitations of this study include its retrospective nature and cohort quality. Furthermore, VSLM lesions were intentionally mirrored to one side of the brain. Although this method is regularly used in stroke studies, the study design assumes that lesions and neurological deficits are distributed equally in both hemispheres.

## Conclusion

This study demonstrates that BBH risk is influenced by both spatial factors (such as target location) and non-spatial factors (such as patient and tumor characteristics). Specifically, the presence of HGG and older patient age increase the risk of BBH. Additionally, target locations in the posterior portions of the putamen, globus pallidus, and the posterior limb of the internal capsule are linked to a higher risk of hemorrhage, particularly symptomatic BBH with somatosensory deficit.

## Data Availability

The data that support the findings of this study are available from the corresponding author upon reasonable request.

## Abbreviations

BB: brain biopsy
BBH: brain biopsy related hemorrhages
BFR: bone fiducial registration
CT: computer tomography
DY: diagnostic yield
EP: entry point
HGG: high grade glioma
LGG: low grade glioma
LSR: laser surface registration
MRI: magnetic resonance imaging
VSLM: Voxel-based lesion symptom mapping
TP: target point

## References

[1] Avval AH, Banerjee S, Zielke J, Kann BH, Mueller S, Rauschecker AM (2025) Applications of artificial intelligence and advanced imaging in pediatric diffuse midline glioma. Neuro Oncol 27:1419–1433. 10.1093/NEUONC/NOAF058 40037540 10.1093/neuonc/noaf058 PMC12309720

[2] Barkley AS, Sullivan LT, Gibson AW, Camacho D, Barber JK, Ko AL, Silbergeld DL, Ravanpay AC (2020) Stereotactic brain biopsy hemorrhage risk factors and implications for postoperative care at a single institution: an argument for postoperative imaging. World Neurosurg 144:e807–e812. 10.1016/J.WNEU.2020.09.084 32956884 10.1016/j.wneu.2020.09.084

[3] Bates E, Wilson SM, Saygin AP, Dick F, Sereno MI, Knight RT, Dronker NF (2003) Voxel-based lesion–symptom mapping. Nature Neuroscience 2003 6:5 6:448–450. 10.1038/nn1050 10.1038/nn1050 12704393

[4] Callovini GM, Sherkat S, Sperduti I, Crispo F, Raus L, Gazzeri R, Telera S (2021) Hemorrhagic attitude in frameless and Frame-Based stereotactic biopsy for Deep-Seated primary central nervous system lymphomas in immunocompetent patients: A multicentric analysis of the last Twenty years. World Neurosurg 149:e1017–e1025. 10.1016/J.WNEU.2021.01.035 33476784 10.1016/j.wneu.2021.01.035

[5] Chakhoyan A, Yao J, Leu K, Pope WB, Salamon N, Yong W, Lai A, Nghiemphu PL, Everson RG, Prins RM, Liau LM, Nathanson DA, Cloughesy TF, Ellingson BM (2019) Validation of vessel size imaging (VSI) in high-grade human gliomas using magnetic resonance imaging, image-guided biopsies, and quantitative immunohistochemistry. Sci Rep 9. 10.1038/S41598-018-37564-W 10.1038/s41598-018-37564-w PMC6391482 30808879

[6] Elias WJ, Sansur CA, Frysinger RC (2009) Sulcal and ventricular trajectories in stereotactic surgery. J Neurosurg 110:201–207. 10.3171/2008.7.17625 18821828 10.3171/2008.7.17625

[7] Ellingson BM, Cloughesy TF, Pope WB, Zaw TM, Phillips H, Lalezari S, Nghiemphu PL, Ibrahim H, Naeini KM, Harris RJ, Lai A (2012) Anatomic localization of O6-methylguanine DNA methyltransferase (MGMT) promoter methylated and unmethylated tumors: a radiographic study in 358 de Novo human glioblastomas. NeuroImage 59:908–916. 10.1016/J.NEUROIMAGE.2011.09.076 22001163 10.1016/j.neuroimage.2011.09.076

[8] Ellingson BM, Lai A, Harris RJ, Selfridge JM, Yong WH, Das K, Pope WB, Nghiemphu PL, Vinters HV, Liau LM, Mischel PS, Cloughesy TF (2013) Probabilistic radiographic atlas of glioblastoma phenotypes. AJNR Am J Neuroradiol 34:533–540. 10.3174/AJNR.A3253 22997168 10.3174/ajnr.A3253 PMC7964888

[9] Fawaz R, Bensemain M, Mokhtari K, Nichelli L, Mathon B (2025) Evaluating hemorrhagic risks using intratumoral susceptibility signals in stereotactic brain biopsies. Neurosurg Rev 48:1–10. 10.1007/S10143-025-03603-0/FIGURES/4 10.1007/s10143-025-03603-0 40434478

[10] Field M, Witham TF, Flickinger JC, Kondziolka D, Lunsford LD (2001) Comprehensive assessment of hemorrhage risks and outcomes after stereotactic brain biopsy. J Neurosurg 94:545–551. 10.3171/JNS.2001.94.4.0545 11302651 10.3171/jns.2001.94.4.0545

[11] Friston KJ, Ashburner JT, Kiebel SJ, Nichols TE, Penny WD (2007) Statistical parametric mapping. The Analysis of Functional Brain Images

[12] Grossman R, Sadetzki S, Spiegelmann R, Ram Z (2005) Haemorrhagic complications and the incidence of asymptomatic bleeding associated with stereotactic brain biopsies. Acta Neurochir (Wien) 147:627–631. 10.1007/S00701-005-0495-5 15821863 10.1007/s00701-005-0495-5

[13] Hu LS, Eschbacher JM, Dueck AC, Heiserman JE, Liu S, Karis JP, Smith KA, Shapiro WR, Pinnaduwage DS, Coons SW, Nakaji P, Debbins J, Feuerstein BG, Baxter LC (2012) Correlations between perfusion MR imaging cerebral blood volume, microvessel quantification, and clinical outcome using stereotactic analysis in recurrent high-grade glioma. AJNR Am J Neuroradiol 33:69–76. 10.3174/AJNR.A2743 22095961 10.3174/ajnr.A2743 PMC7966183

[14] Kreth FW, Muacevic A, Medele R, Bise K, Meyer T, Reulen HJ (2001) The risk of haemorrhage after image guided stereotactic biopsy of intra-axial brain tumours–a prospective study. Acta Neurochir (Wien) 143:539–546. 10.1007/S007010170058 11534670 10.1007/s007010170058

[15] Kulkarni AV, Guha A, Lozano A, Bernstein M (1998) Incidence of silent hemorrhage and delayed deterioration after stereotactic brain biopsy. J Neurosurg 89:31–35. 10.3171/JNS.1998.89.1.0031 9647169 10.3171/jns.1998.89.1.0031

[16] Lara-Almunia M, Hernandez-Vicente J (2020) Symptomatic intracranial hemorrhages and frame-based stereotactic brain biopsy. Surg Neurol Int 11:218. 10.25259/SNI_102_2020 32874721 10.25259/SNI_102_2020 PMC7451146

[17] Li H, Zheng C, Rao W, Sun J, Yu X, Zhang J (2022) The risk factors of hemorrhage in stereotactic needle biopsy for brain lesions in a large cohort: 10 years of experience in a single center. Chin Neurosurg J 8:40. 10.1186/S41016-022-00307-Y 36494749 10.1186/s41016-022-00307-y PMC9732999

[18] Lozano AM, Gildenberg PL, Tasker RR (2009) Textbook of stereotactic and functional neurosurgery. Springer Science & Business Media

[19] Machetanz K, Grimm F, Schuhmann M, Tatagiba M, Gharabaghi A, Naros G (2021) Time efficiency in stereotactic Robot-Assisted surgery: an appraisal of the surgical procedure and surgeon’s learning curve. Stereotact Funct Neurosurg 99:25–33. 10.1159/000510107 33017833 10.1159/000510107

[20] Machetanz K, Grimm F, Wang S, Bender B, Tatagiba M, Gharabaghi A, Naros G (2021) Patient-to-robot registration: the fate of robot-assisted stereotaxy. Int J Med Rob Comput Assist Surg 17. 10.1002/rcs.2288 10.1002/rcs.2288 34036749

[21] Machetanz K, Grimm F, Wang S, Schuhmann MU, Tatagiba M, Gharabaghi A, Naros G (2022) Rediscovery of the transcerebellar approach: improving the risk-benefit ratio in robot-assisted brainstem biopsies. Neurosurg Focus 52:E12. 10.3171/2021.10.FOCUS21359 34973665 10.3171/2021.10.FOCUS21359

[22] Malone H, Yang J, Hershman DL, Wright JD, Bruce JN, Neugut AI (2015) Complications following stereotactic needle biopsy of intracranial tumors. World Neurosurg 84:1084–1089. 10.1016/J.WNEU.2015.05.025 26008141 10.1016/j.wneu.2015.05.025

[23] McGirt MJ, Woodworth GF, Coon AL, Frazier JM, Amundson E, Garonzik I, Olivi A, Weingart JD (2005) Independent predictors of morbidity after image-guided stereotactic brain biopsy: a risk assessment of 270 cases. J Neurosurg 102:897–901. 10.3171/JNS.2005.102.5.0897 15926716 10.3171/jns.2005.102.5.0897

[24] Mizobuchi Y, Nakajima K, Fujihara T, Matsuzaki K, Mure H, Nagahiro S, Takagi Y (2019) The risk of hemorrhage in stereotactic biopsy for brain tumors. J Med Invest 66:314–318. 10.2152/JMI.66.314 31656296 10.2152/jmi.66.314

[25] Muroya Y, Suzuki K, Nagasaka S, Nakano Y, Yamamoto J (2022) Primary central nervous system lymphoma of the third ventricle with intra-tumoral hemorrhage: A case report and literature review. Oncol Lett 25. 10.3892/OL.2022.13633 10.3892/ol.2022.13633 PMC9811644 36644156

[26] Naros G, Machetanz K, Grimm F, Roser F, Gharabaghi A, Tatagiba M (2021) Framed and non-framed robotics in neurosurgery: A 10-year single-center experience. Int J Med Rob Comput Assist Surg 17. 10.1002/rcs.2282 10.1002/rcs.2282 34030218

[27] Park MJ, Kim HS, Jahng GH, Ryu CW, Park SM, Kim SY (2009) Semiquantitative assessment of intratumoral susceptibility signals using Non-Contrast-Enhanced High-Field High-Resolution susceptibility-Weighted imaging in patients with gliomas: comparison with MR perfusion imaging. AJNR Am J Neuroradiol 30:1402. 10.3174/AJNR.A1593 19369602 10.3174/ajnr.A1593 PMC7051537

[28] Patel KS, Carter BS, Chen CC (2018) Role of biopsies in the management of intracranial gliomas. Prog Neurol Surg 30:232–243. 10.1159/000464439 29241178 10.1159/000464439

[29] Pouratian N, Sheth SA (2020) Stereotactic and functional neurosurgery. Springer Nature

[30] Quick-Weller J, Lescher S, Bruder M, Dinc N, Behmanesh B, Seifert V, Weise L, Marquardt G (2016) Stereotactic biopsy of brainstem lesions: 21 years experiences of a single center. J Neurooncol 129:243–250. 10.1007/S11060-016-2166-1 27291894 10.1007/s11060-016-2166-1

[31] Sakata A, Okada T, Yamamoto A, Kanagaki M, Fushimi Y, Dodo T, Arakawa Y, Takahashi JC, Miyamoto S, Togashi K (2015) Primary central nervous system lymphoma: is absence of intratumoral hemorrhage a characteristic finding on MRI? Radiol Oncol 49:128. 10.1515/RAON-2015-0007 26029023 10.1515/raon-2015-0007 PMC4387988

[32] Shakal AAS, Mokbel EAH (2014) Hemorrhage after stereotactic biopsy from intra-axial brain lesions: incidence and avoidance. J Neurol Surg Cent Eur Neurosurg 75:177–182. 10.1055/S-0032-1325633 10.1055/s-0032-1325633 23526202

[33] Sun X, Li S, Ma C, Fang W, Jing X, Yang C, Li H, Zhang X, Ge C, Liu B, Li Z (2024) Glioma subtype prediction based on radiomics of tumor and peritumoral edema under automatic segmentation. Scientific Reports 2024 14:1 14:1–10. 10.1038/s41598-024-79344-9 10.1038/s41598-024-79344-9 PMC11551193 39523433

[34] Takano K, Kinoshita M, Takagaki M, Sakai M, Tateishi S, Achiha T, Hirayama R, Nishino K, Uchida J, Kumagai T, Okami J, Kawaguchi A, Hashimoto N, Nakanishi K, Imamura F, Higashiyama M, Yoshimine T (2016) Different Spatial distributions of brain metastases from lung cancer by histological subtype and mutation status of epidermal growth factor receptor. Neuro Oncol 18:716–724. 10.1093/NEUONC/NOV266 26519739 10.1093/neuonc/nov266 PMC4827044

[35] Tanji M, Mineharu Y, Sakata A, Okuchi S, Fushimi Y, Oishi M, Terada Y, Sano N, Yamao Y, Arakawa Y, Yoshida K, Miyamoto S (2022) High intratumoral susceptibility signal grade on susceptibility-weighted imaging: a risk factor for hemorrhage after stereotactic biopsy. J Neurosurg 138:120–127. 10.3171/2022.4.JNS212505 35561695 10.3171/2022.4.JNS212505

[36] Tzourio-Mazoyer N, Landeau B, Papathanassiou D, Crivello F, Etard O, Delcroix N, Mazoyer B, Joliot M (2002) Automated anatomical labeling of activations in SPM using a macroscopic anatomical parcellation of the MNI MRI single-subject brain. NeuroImage 15:273–289. 10.1006/nimg.2001.0978 11771995 10.1006/nimg.2001.0978

[37] Warnick RE, Longmore LM, Paul CA, Bode LA (2003) Postoperative management of patients after stereotactic biopsy: results of a survey of the AANS/CNS section on tumors and a single institution study. J Neurooncol 62:289–296. 10.1023/A:1023315206736/METRICS 12777081 10.1023/a:1023315206736

[38] Wen PY, Packer RJ (2021) The 2021 WHO classification of tumors of the central nervous system: clinical implications. Neuro Oncol 23:1215. 10.1093/NEUONC/NOAB120 34185090 10.1093/neuonc/noab120 PMC8328017

[39] Williams JR, Young CC, Vitanza NA, Mcgrath M, Feroze AH, Browd SR, Hauptman JS (2020) Progress in diffuse intrinsic Pontine glioma: advocating for stereotactic biopsy in the standard of care. Neurosurg Focus 48. 10.3171/2019.9.FOCUS19745 10.3171/2019.9.FOCUS19745 31896081

[40] Wu O, Cloonan L, Mocking SJT, Bouts MJRJ, Copen WA, Cougo-Pinto PT, Fitzpatrick K, Kanakis A, Schaefer PW, Rosand J, Furie KL, Rost NS (2015) The role of acute lesion topography in initial ischemic stroke severity and long-term functional outcomes. Stroke 46:2438. 10.1161/STROKEAHA.115.009643 26199314 10.1161/STROKEAHA.115.009643 PMC4550548

[41] Wu S, Wang J, Liu W, Hu F, Zhao K, Jiang W, Lei T, Shu K (2021) The role of surgical resection in primary central nervous system lymphoma: a single-center retrospective analysis of 70 patients. BMC Neurol 21. 10.1186/S12883-021-02227-3 10.1186/s12883-021-02227-3 PMC8112018 33975554

[42] Yamashita K, Hiwatashi A, Togao O, Kikuchi K, Momosaka D, Hata N, Akagi Y, Suzuki SO, Iwaki T, Iihara K, Honda H (2019) Differences between primary central nervous system lymphoma and glioblastoma: topographic analysis using voxel-based morphometry. Clin Radiol 74:816e1. 816.e8 10.1016/j.crad.2019.06.017 31400805

[43] Zanello M, Carron R, Peeters S, Gori P, Roux A, Bloch I, Oppenheim C, Pallud J (2021) Automated neurosurgical stereotactic planning for intraoperative use: a comprehensive review of the literature and perspectives. Neurosurg Rev 44:867–888. 10.1007/S10143-020-01315-1 32430559 10.1007/s10143-020-01315-1

[44] Zanello M, Debacker C, Moiraghi A, Peeters S, Roux A, Deboeuf L, Parraga E, Dezamis E, Chrétien F, Oppenheim C, Pallud J (2023) Use of MR signal intensity variations to highlight structures at risk along brain biopsy trajectories. J Neurosurg 140:116–126. 10.3171/2023.5.JNS23263 37548577 10.3171/2023.5.JNS23263

[45] Zanello M, Roux A, Debacker C, Peeters S, Edjlali-Goujon M, Dhermain F, Dezamis E, Oppenheim C, Lechapt-Zalcman E, Harislur M, Varlet P, Chretien F, Devaux B, Pallud J (2021) Postoperative intracerebral haematomas following stereotactic biopsies: poor planning or poor execution? Int J Med Robot 17. 10.1002/RCS.2211 10.1002/rcs.2211 33345461

[46] Zanello M, Roux A, Senova S, Peeters S, Edjlali M, Tauziede-Espariat A, Dezamis E, Parraga E, Zah-Bi G, Harislur M, Oppenheim C, Sauvageon X, Chretien F, Devaux B, Varlet P, Pallud J (2021) Robot-Assisted stereotactic biopsies in 377 consecutive adult patients with supratentorial diffuse gliomas: diagnostic Yield, Safety, and postoperative outcomes. World Neurosurg 148:e301–e313. 10.1016/J.WNEU.2020.12.127 33412330 10.1016/j.wneu.2020.12.127

[47] Zhang Y, Kimberg DY, Coslett HB, Schwartz MF, Wang Z (2014) Multivariate lesion-symptom mapping using support vector regression. Hum Brain Mapp 35:5861–5876 1002/HBM.22590;PAGEGROUP:STRING:PUBLICATION 25044213 10.1002/hbm.22590 PMC4213345

[48] Zrinzo L, Van Hulzen ALJ, Gorgulho AA, Limousin P, Staal MJ, De Salles AAF, Hariz MI (2009) Avoiding the ventricle: A simple step to improve accuracy of anatomical targeting during deep brain stimulation - Clinical Article. J Neurosurg 110:1283–1290. 10.3171/2008.12.JNS08885 19301961 10.3171/2008.12.JNS08885

